# Acetanilid (or Antifebrin)1Read before the semi-annual meeting of the Pennsylvania State Veterinary Medical Association, October, 1896.

**Published:** 1897-01

**Authors:** F. S. Allen

**Affiliations:** Philadelphia


### ACETANILID (OR ANTIFEBRIN).[1]

BY F. S. ALLEN, M.D., D.V.S.,
PHILADELPHIA.

ACETANILID was first employed in medicine by Cahn and Hepp. It is a white, crystalline substance readily soluble in water. When taken into the mouth it causes a slight burning sensation. It is almost if not quite tasteless, and therefore is not disagreeable to take. Dr. Wood describes it as bitter and piquant in taste. Acetanilid has long been known to chemists, and is the product of action of glacial acetic acid upon anilin. Antifebrin is a name given to this drug by a certain firm having exclusive control of all manufactured and sold under that name. Antifebrin commands a higher price. Therefore, in prescription-writing we should write for acetanilid as a matter of economy, since it gives equally good results.

Drs. Hare and Wood, of Philadelphia, have conducted numerous experiments, both physiologically and clinically, with this drug, and from their published works I have drawn much of the material for this paper.

*Physiological action.* 1. Upon the nervous system acetanilid acts as a sedative, quieting the sensory portions of the nerves and spinal cord. In large, poisonous doses it produces anæsthesia, a total loss of reflex action, and motor and sensory paralysis. Dr. Hare claims that the sensory side of the spinal cord and sensory nerves are first affected, and later the motor apparatus, and that the muscles are influenced by the poison only indirectly.

2. On the circulation it has but little effect, unless used in large and poisonous doses. In such cases there is an immediate fall of arterial pressure, with a diminution in the size of pulse-wave, and all the evidence of cardiac and circulatory depression, although death is said to result from respiratory failure. The cause of this fall of blood-pressure is said to be due to its direct depressing action upon the heart, associated with its effects on the vasomotor system, as asphyxia causes no rise in pressure. In medicinal doses there may be either an increased or diminished pulse-rate. Its action upon the blood in large doses is said to be marked, causing it to become brownish-red in color, decreasing its ozonizing and

[1] Read before the semi-annual meeting of the Pennsylvania State Veterinary Medical Association, October, 1896.

oxygen-carrying power, and finally reducing largely the hæmoglobin to methæmoglobin.

Its action upon the red blood-corpuscles is undetermined, some claiming they are disorganized, others that they are not. Dr. Hare claims that in moderately large, poisonous doses the drug does not affect the corpuscles; but if continued for some days, or if a very large dose be used at one time, corpuscular destruction occurs, free hæmoglobin appears in the urine, the normal alkalinity of the blood is lessened, and the urine becomes dark-brown in color.

3. Acetanilid given in small doses is said to have no action upon the respiration, but in large, poisonous doses it at once produces a more rapid breathing, which soon becomes labored. Death is said to result from these large doses by paralysis of the respiratory centres, and the primary causes are the alteration in the blood, the want of oxygenation of the tissues, and at the same time the depressing action of the drug itself. Baker asserts that the drug also paralyzes the peripheral motor nerves.

4. Dr. Hare claims that the drug when given in full medicinal doses lowers the normal temperature or may produce no change. In poisonous doses it produces a decrease in temperature depending on the amount employed, and may produce collapse and rigors. On fevers he claims that it acts as a powerful and fairly constant antipyretic, lowering the fever by decreasing heat-production, and increasing heat-dissipation, heat-production being the function most affected.

As to its action upon the kidney there is much dispute, but it is agreed that the excretion of urine is increased, and from excessive doses the urine becomes dark, it is said, from broken-down blood-coloring matter. Elimination takes place through the kidneys as paraphenol sulphate.

It is slightly antiseptic when used freely. I have used it but little, preferring to use others that are better, as iodoform, etc.

Toxic changes from prolonged use are claimed to occur, such as congestion of the liver, kidney, and spleen, and from poisonous doses it is said clots may be found in the cardiac cavity and a progressive decrease in the red blood-corpuscles.

*Therapeutical actions.* In the use of the drug much depends upon the condition of the animal and the disease from which it suffers. Although a powerful antipyretic in some diseases, it has apparently little or no action or influence upon the temperature, the duration, or general course of the disease. In some cases I have known the temperature to continue to rise when given often in full doses, and on change of treatment the temperature would fall. This was noticeable in typhoid conditions. Acetanilid, I find in sthenic fevers, is one of our most reliable drugs, reducing the temperature, and it would seem to often shorten their duration. In debilitating or asthenic diseases it has little or no effect on the temperature or general course of the disease. In these diseases, when used, there is great depression and danger of collapse. Good results are claimed from its use in rheumatism, epilepsy, gastralgia, and sciatica, etc.

(To be continued.)

